# Analysis of factors influencing the frequency of primary care visits among diabetic patients in two provinces in China

**DOI:** 10.1186/s12889-019-7591-6

**Published:** 2019-09-13

**Authors:** Jinwen Wang, Huijuan Zuo, Xiaorong Chen, Lei Hou, Jixiang Ma

**Affiliations:** 1 Beijing Anzhen Hospital, Beijing Institute of Heart Lung and Blood Vessel Disease, Capital Medical University, Beijing, 100029 People’s Republic of China; 2 Department of Chronic Non-communicable Diseases Prevention, Chinese Center for Disease Control and Prevention, No.27 Road Nanwei, Beijing, 100050 Xicheng District China

**Keywords:** Type 2 diabetes, Frequent attendance, Community, Utility of care

## Abstract

**Background:**

Community health services have played an important role in the prevention and control of diabetes in China. The aims of this study were to examine the frequency of visits to community clinics for diabetic care services, to assess factors correlated with infrequent primary care visits and to identify barriers to regular follow-up visits for urban and rural patients.

**Methods:**

Between October 2014 and November 2014, data were collected from 17 communities in two cities and four townships located in Shandong and Jiangsu Provinces in China. A total of 1598 diabetic patients aged 18 years or older who were registered with a primary health station in local communities were selected by simple random sampling. Each participant was required to complete an interviewer-led questionnaire. Univariate and multivariate analyses were used to identify significant factors for infrequent visitor status using multivariable logistic regression analysis.

**Results:**

After being clearly informed of the study protocol, 1508/1598 (94.4%) patients agreed to participate in this survey. Among the 1508 subjects (mean age 64.4 ± 10.6), 683 (45.3%) were classified as infrequent visitors. The following were significant factors determining infrequent visitor status: urban residence, lack of health insurance, per-capita household income< 20,000 (yuan), lack of telephone follow-up and lack of household visit. From the patients’ perspectives, the reasons for infrequent visits among urban patients included drug scarcity and longer travel time to clinics. For rural patients, worries about medical expenses and drug scarcity were the most common barriers to clinic visits.

**Conclusion:**

Determinants of infrequent community visits in diabetes patients include urban residence, lower household income, lack of health insurance, lack of telephone follow-up and lack of household visit services. Strategies aimed at enhancing the utilization of community health care should be implemented in China.

## Background

Diabetes has become a major public health problem throughout the world. In China, both urbanization and a trend towards Westernized lifestyles have resulted in an epidemic of type 2 diabetes mellitus (T2DM). It has been reported that the prevalence of diabetes is 9.7% in China, accounting for 92.4 million adults with diabetes [1]. Diabetes mellitus not only affects the physical well-being of patients but also leads to serious health complications, which has caused a heavy economic burden in China [2]. There is evidence that use of care services among diabetic patients is vital to overall self-care of diabetes and to optimization of disease-related outcomes. Although diabetes patients in urban regions have better services utilization than those in rural area, lack of knowledge for disease management and delayed treatment are also common in Chinese cities [3]. Recent studies reported that there is low proportion of diabetic patients receiving recommended annual eye and foot examinations in China. The prevalence of diabetic retinopathy in patients with T2DM in Tai’an City in Shandong province is 9.01% [4].

Community-based strategies have gained increasing attention and represent a promising area of development in chronic disease care in developing counties [5, 6]. Several systematic reviews suggest that interventions by community health workers (CHWs) appear to be effective when compared with alternatives and are cost-effective for the management of diabetes [7, 8]. Community-based medical and health organizations were expected to play an important role in diabetes prevention and control in China. Government has formulated a series of polices to encourage the development of primary health care program [9]. Primary care medical settings, such as community health service centres or community clinics, were expected to be the main gatekeepers for the management of diabetes and other chronic diseases in China [10].

Chinese guidelines for the management of non-communicable diseases recommend that patients with diabetes should see their community physician at least once every 1 to 3 months and that patients with uncontrolled diabetes should increase the frequency of clinical visits [11]. Patients were encouraged to participate in regular community clinic visits for medicine prescription, examination, and diabetes education. Furthermore, some CHWs will implement telephone follow-up or home visits towards the self-care of diabetes patients. Local government also implemented strategies to improve the use of diabetic care in community. Shandong and Jiangsu Provinces lies in northern China and southern China, respectively. In recent years, Jiangsu and Shandong governments increased their payment to community for diabetes education. Electronic health record system was used in primary health station to improve the capacity of CHWs. Non-financial incentives including regular training and opportunities for professional development were included as components of community health program in Jiangsu. However, limited studies have been specifically designed to evaluate the current frequency of primary care visits among diabetic adults in China. The aims of this study were to examine the frequency of visits to community physicians for diabetic care services, to assess factors correlated with infrequent primary care visits and to identify barriers to regular follow-up visits for urban and rural patients.

## Methods

The cross-sectional survey was conducted in Shandong and Jiangsu Provinces in China between October 2014 and November 2014. Jiangsu and Shandong are among the most prosperous and important provinces in China, with a share of 20% of the national Gross Domestic Product (GDP). First, we selected 2 urban districts (Qingdao city, Wuxi city) and 4 townships (Rushan, Qiyuan, Lianshui, and Sheyang townships) from Jiangsu and Shandong Provinces. Cities and townships were selected by the local program manager. Criteria for city and township selection include: security, feasibility for travel, and equipped with electronic health records system. Second, 17 communities were chosen by simple random sampling from hundreds communities in the two cities and four townships. Participants were eligible for this study if they were between 18 and 75 years old, have been registered with primary health stations in local communities at least 1 year and have been diagnosed with type 2 Diabetes. A random sample of 90–100 diabetes patients were drawn from electronic health records provided by the primary health station in local community. Allocation will be done by computer generated randomization. A total of 1598 diabetic patients were enrolled in the survey. After being clearly informed of the study protocol, 1508/1598 (89.6%) patients agreed to participate in this survey. Written informed consent was obtained from participants, each of whom was required to complete an interviewer-led questionnaire. Trained community physicians served as interviewers. Except for oral reports, detailed information regarding the medical histories of all participants was provided by the community health service station of each community.

A structured questionnaire was developed by a team of researchers at the Capital Medical University in China. Content finalization was achieved through a literature and policy document review. The survey questionnaire was pilot-tested on 30 diabetic volunteers in the community of Jiangsu to determine the participants’ level of comprehension. The results of the pilot study have not been included in this paper.

The questionnaire concerned demographic information, frequency of visits, and reasons for being unwilling to visit primary care clinics. Demographic information consisted of gender, age, place of residence, occupation, household income, education level, and insurance type. Family history of coronary disease was defined as cardiovascular disease in a first degree male relative < 55 and female relative < 65 years of age.

The frequency of visits was assessed during the interview by asking people “How many times did you visit your general physician (GP) for diabetic care in the previous 12 months?” The answers were classified into one of five categories ranging from “never” to “more than 12 times per year”. For this analysis, infrequent visitors were defined as subjects who visited the community clinics for diabetes care three or fewer times per year [12].

Statistical analyses were conducted using SPSS statistical software (version 18.0; SPSS, Inc., Chicago, IL, USA). A *P* value < 0.05 was considered to indicate statistical significance. Descriptive statistics were used to present means, standard deviations, and percentages. We used the t-test and chi-square test to identify differences in continuous data and categorical variables, respectively, between frequent and infrequent visitors. Univariate and multivariate analyses were used to find significant factors for infrequent visitor status using multivariable logistic regression analysis. Adjusted odds ratios and the corresponding 95% confidence intervals (CIs) were calculated for each independent variable.

## Results

The frequency of primary care visits for diabetic patients in the previous year are shown in Table 1. The majority of patients visited clinics four or more times, with 31.1% visiting 4–6 times, 11.9% visiting 7–12 times, and 11.7% visiting 12 or more times. One hundred and seventy-four patients (11.5%) had never visited the community clinics, and 509 patients (33.7%) visited the community clinic 1–3 times in the previous year.

**Table 1:** Descriptive Statistics for frequency of community clinic visits

Frequency of community clinic visits		%	*N*
Infrequency of attendance	0 time	11.5	174
	1~3 times	33.7	509
Frequency of attendance	4~6 times	31.1	469
	7~12 times	11.9	179
	> 12 times	11.7	177

The baseline characteristics of the participants who participated in the survey are presented in Table 2. Among the 1508 adults with a clinical diagnosis of T2DM included in the analysis, 54.7% were frequent visitors and 45.3% were infrequent visitors. Compared with frequent visitors, a significant proportion of infrequent visitors were urban residents, had a lower prevalence of hypertension, had a lower household income, and had a lack of health insurance. Infrequent visitors received less telephone follow-up and fewer home visits than did frequent visitors. Regarding gender and age, no significant differences were found between the two groups.

**Table 2:** Differences in characteristics of participants between infrequent and frequent visitors

Characteristics	Total (*n* = 1508)	Frequent visitor *n* = 825	Infrequent visitor *n* = 683	*P* value
Urban/rural, *n* (%)				0.001
Urban	748 (49.6)	378 (45.8)	370 (54.2)	
Rural	760 (50.4)	447 (54.2)	313 (45.8)	
Gender, *n* (%)				0.493
Male	605 (40.1)	324 (39.3)	281 (41.1)	
Female	903 (59.9)	501 (60.7)	402 (58.9)	
Age, *n* (%)				
Mean age, *M* (*SD*)	64.4 ± 10.6	64.3 ± 10.3	64.2 ± 10.9	0.873
≤ 55 yr	266 (17.6)	134 (16.2)	132 (19.3)	0.175
56-65 yr	534 (35.4)	305 (37.0)	229 (33.5)	
≥ 66 yr	708 (46.9)	386 (46.8)	322 (47.1)	
Years since diagnosis of diabetes ≥10 yr., *n* (%)	436 (28.9)	236 (28.6)	200 (29.3)	0.776
Family history of coronary disease, *n* (%)	184 (12.2)	110 (13.3)	74 (10.8)	0.198
Medical history, *n* (%)				
Hyperlipidemia	556 (36.9)	309 (37.5)	247 (36.2)	0.592
Hypertension	822 (54.5)	479 (58.1)	343 (50.2)	0.002
Myocardial infarction	27 (1.8)	17 (2.1)	10 (1.5)	0.439
Stenting	10 (0.7)	5 (0.6)	5 (0.7)	0.763
CABG	10 (0.7)	4 (0.5)	6 (0.9)	0.363
Stroke	108 (7.2)	64 (7.8)	42 (6.4)	
Educational level, *n* (%)				0.336
Intermediate school or lower	1264 (83.8)	699 (84.7)	565 (82.7)	
High school	179 (11.9)	96 (11.6)	83 (12.2)	
≥ College graduate	65 (4.4)	30 (3.7)	35 (5.1)	
Per capita household income (yuan), *n (%)*				0.011
< 5000	434 (28.8)	223 (27)	211 (30.9)	
5000~19,999	632 (41.9)	349 (42.3)	283 (41.4)	
≥ 20,000	394 (26.1)	238 (28.8)	156 (22.8)	
Refused to answer	48 (3.2)	15 (1.8)	33 (4.8)	
Lack Health insurance in the past 12 months, *n (%)*	21 (1.4)	3 (0.36)	18 (2.6)	< 0.001
Health care service				
Household visit	467 (31.0)	354 (42.9)	113 (16.5)	< 0.001
Telephone follow-up	739 (49.0)	472 (57.2)	267 (39.1)	< 0.001

The results of multivariable backward stepwise logistic regression analyses are shown in Table 3. Based on the entry criteria mentioned in the statistical analysis section, the variables entered into the multivariable logistic regression analysis included urban residence (yes/no), hypertension (yes/no), lack of health insurance (yes/no), per-capita household income (< 5000/5000–2000/> 20,000(yuan)), household visit (yes/no) and telephone follow-up (yes/no). As shown in Table 3, the variables positively associated with infrequent visitor status were urban residence (OR = 1.696, [1.293, 2.224], *p* <  0.001), lack of health insurance in the previous 12 months (OR = 6.854, [1.992, 23.578], *p* = 0.002), telephone follow-up (OR = 0.507, [0.400 to 0.643], p <  0.001), and household visit (OR = 0.313 [0.241, 0.407], *p* <  0.001). In addition infrequent visitor were more likely to have a per capita household income < 5000 yuan (OR = 2.621 [1.859, 3.696]), 5000–20,000 yuan (OR = 2.008 [1.487, 2.712]) than patients have a per capita household income > 20,000 yuan.

**Table 3:** Significant factors contributing to infrequent visitor status based on the multiple logistic regression analyses^†^

Variables	OR (95% CI)	*P* value
Urban vs. rural	1.696 (1.293, 2.224)	< 0.001
Lack health insurance, (yes vs. no)	6.854 (1.992, 23.578)	0.002
Household visit (yes vs. no)	0.313 (0.241, 0.407)	< 0.001
Telephone follow-up (yes vs. no)	0.507 (0.400, 0.643)	< 0.001
Per capita household income		
> 20,000 yuan	─	
5000–20,000 yuan	2.008 (1.487, 2.712)	< 0.001
< 5000 yuan	2.621 (1.859,3.696)	< 0.001

*OR* Odds ratio, *CI* confidence interval

^†^ The backward stepwise method was used, which was adjusted for age, gender, educational level, history of hypertension, myocardial infarction, stent, CABG, and stroke

Table 4 shows the significant factors contributing to infrequent visitor status stratified by urban/rural area. Rural adults with infrequent visitor status were more likely to lack health insurance (OR = 9.317, *p* = 0.008) and have a per capita household income < 5000 yuan (OR = 1.725, *p* <  0.001) than patients have a per capita household income > 20,000 yuan. In addition, rural patients received regular household visit (OR = 0.278, p <  0.001) and telephone follow-up (OR = 0.432, p <  0.001) tended not to be infrequent visitors. A similar trend can be seen in urban patients. That is, all of the above significant factors in rural subjects were also significant in urban residents, but they were in different odds. The ORs in urban residents indicated that lack of health insurance and a per capita household income < 5000 yuan increased the odds of infrequent visitor status by 4.750 (*P* = 0.004) and 2.639 (*P* <  0.001) times, respectively; while household visit and telephone follow-up decreased the risk of being infrequent visitor status (ORs = 0.341 and 0.604 respectively). (Additional file 1: Table S1a to S1c show the multivariate analysis with all variables.)

**Table 4:** Significant factors contributing to infrequent visitor status based on the multiple logistic regression analyses stratified by urban/rural area^†^

Variables	Urban		Rural	
	OR (95% CI)	*P* value	OR (95% CI)	*p* value
Lack health insurance, (yes vs. no)	4.750 (1.334, 16.912)	0.027	9.317 (1.768, 49.091)	0.008
Household visit (yes vs. no)	0.341 (0.222, 0.523)	< 0.001	0.278 (0.198, 0.391)	< 0.001
Telephone follow-up (yes vs. no)	0.604 (0.438, 0.834)	0.004	0.432 (0.303, 0.615)	< 0.001
Per capita household income				
> 20,000 yuan	─		─	
5000–20,000 yuan	2.329 (1.638, 3.313)	< 0.001	1.215 (1.027, 2.318)	< 0.001
< 5000 yuan	2.639 (1.616, 4.310)	< 0.001	1.725 (1.196, 3.299)	< 0.001

*OR* Odds ratio, *CI* Confidence interval

^†^The backward stepwise method was used, which was adjusted for age, gender, educational level, history of hypertension, myocardial infarction, stent, CABG, and stroke

Table 5 shows the reasons for infrequent visits to community clinics. We obtained answers from 379 infrequent visitors (130 urban, 249 rural) in community clinics. A number of factors were mentioned to explain infrequent visits. Forty-five urban patients (34.6%) indicated that the scarcity of diabetes medicines in the community clinics was the major reason for infrequent visits. Thirty-four patients (26.2%) mentioned a longer travel time to community clinics as their reason, while 6.2% claimed that the quality of care provided by CHWs was poor. The most common anxiety expressed by rural patients was worrying about the medical expenses and drug scarcity.

**Table 5:** Reasons for unwilling to visit primary care clinics for diabetes patients

Reasons	*N* (%)
Urban (*n* = 130)	
Many diabetes medicines were scarce in community	45 (34.6)
It is a long distance from home to community clinics	34 (26.2)
The quality of care provided by CHWs was poor	8 (6.2)
Rural (*n* = 249)	
Worry about the medical expenses	86 (34.5)
Many diabetes medicines were scarce in community	72 (28.9)
The quality of care provided by CHWs was poor	44 (17.7)

## Discussion

Diabetes is not only a common metabolic disorder affecting an increasing number of individuals but also an important cardiovascular risk factor. To attain optimal T2DM health outcomes, patients must participate in self-care management, which includes eating a recommended diet, engaging in regular exercise, monitoring blood glucose, and adhering to their medication regimen [13, 14]. However, poor adherence occurs frequently in diabetes patients, and motivating patients to achieve high self-care adherence is challenging. it has been indicated that CHW-led patient coaching interventions could facilitate the acceptance and effectiveness of self-management and lead to improved outcomes.

Diabetes is by far a serious health challenge in China, where many low-income populations exist. Community services were expected to play a key role in improving the health care level in diabetes patients, especially in low-income populations. A series of projects have been carried out to promote the development of community health services [15]. The community health centre (CHC) was first established in 1997, and thereafter its workforce (CHW) has expanded rapidly during the last decade [16, 17]. The number of CHWs increased from 17,281 in 2003 to 109,734 in 2009, while that of nurses grew from 12,484 to 79,711. However, the results of the present study revealed that the community service for diabetic care was not fully utilized in China. The frequency of nearly one-third of Chinese adults to see their GPs was less than four times per year, which is significantly lower than in Canada, where 90% of seniors reported that they had seen their medical doctors more thanfour times in the past 12 months.

Several studies have suggested that comorbidity in diabetes patients significantly impacts their ability to self-manage and utilize healthcare services [18, 19]. In this study, diabetes patients tended to see doctors less frequently than those in previous reports. Approximately two-thirds of subjects in this study had 1 or more comorbidities, which is lower than the 80 to 90% frequently cited. In the present study, hypertension was the most common comorbid condition, affecting 54.1% of patients. However, Gruneir et al. reported a higher proportion of 79.1% [18]. Therefore a lower prevalence of concordant conditions (such as hypertension, ischaemic heart disease and disorders of lipid metabolism) may partly explain the lower incidence of clinical visit in the present study.

Our study revealed that urban residence, lack of health insurance, a per-capita household income< 20,000 yuan, lack of telephone follow-up and lack of household visits were significant factors in terms of infrequent visitor status. Similar results could be seen in other studies. Lutfiyya et al. reported that adults receiving less than adequate care for diabetes were more likely to be male, less educated, unmarried, economically poorer, inactive, and a smoker [20]. Garvey et al. found that patients receiving current care from an endocrinologist were more likely to report more diabetes visits than those receiving diabetes care from a primary care doctor [21].

The patient’s economic and cultural environment was known to be a major factor in his/her behavioural decisions [22, 23]. Lower socioeconomic status is associated with less clinical visits. It has been reported that the type or availability of health insurance might affect attendance rates [24]. Medical insurance has not yet covered the entire population in China. In this study, 1.4% of patients lacked health insurance in the past 12 months. Our results revealed a higher infrequency visit rate both in the population lacking health insurance and those with low-income, which was also compatible with the findings of previous studies.

Attendance rates have been improved by the use of reminders sent via a short message service or telephone calls [25–27]. In the present study, the frequency of clinic visits was lower if the CHWs had implemented telephone visits or home visits. Telephone visits or home visits could increase trust between CHWs and patients and diminish anxiety, which may improve patients’ willingness to visit community clinics.

Our results indicated that urban diabetic patients were less likely to visit community clinics than were rural patients. Individuals living in urban areas are less likely to be poor and uninsured than those living in rural areas. Compared to rural areas, there are more health care organizations of all kinds in urban areas, as well as more choices among them [28]. Although the government is now pushing ahead with the implementation of the hierarchical medical system, patients are allowed to see doctors at tertiary hospitals without needing an appointment or referral from a primary health station. Therefore, many urban patients visited tertiary hospitals for primary care.

In the present study, the most frequently mentioned factors from the patients’ perspectives were the availability of medication in the community and the poor quality of community health care. A recent study found that both the list and supply of essential medicines could not adequately meet the clinical needs of patients at community health centres in China [29]. In addition, China faced problems with the recruitment of skilled health professionals in the community in recent years [30]. Health workers are more likely to be family physicians or generalists with a broad scope of practice in the community. Patients’ disrespect or an unsatisfactory relationship with the clinician might result in non-attendance. For rural patients, the medicine expenditure was found to have a negative impact on community clinic visits. By 2014, the reimbursement rate was only 60% for expenses for community health services in rural areas, meaning that medical care was a heavy economic burden [31].

We recommend that managers and healthcare policymakers take our results into consideration to develop interventions to increase the utilization of primary health care. First, the government should accelerate the reform of the hierarchical medical system and guide patients to voluntarily see a doctor in primary medical institutions. Second, programme-specific training is required for health care workers to improve the quality of care for diabetes patients. The services that CHWs should provide and the required skill set for those services should be determined. CHWs may require training in preventive medicine, the healthcare system, the importance of primary care, and patient-centred medical home concepts. The government should also expand eligible activities to help communities recruit and retain skilled physicians. Third, healthcare policymakers need to consider various programmes to increase the supply of low-priced drugs and essential medicines for the community, particularly for those vulnerable to medical expenditures. The rural residents should be covered by basic medical insurance, and the reimbursement rate for services performed within the primary health care station should be increased. Forth, the data on telephone follow-up and household visits should be recorded in electronic health records, which are basis of public health service assessment. Financial incentives, whether for allowances, or per diem payment, should be provided for CHWs to encourage motivation for telephone follow-up and household visits.

Our study focused not only on the utilization of community service in diabetes care but also evaluate the detailed reasons. The results of this study may be useful for evaluating primary health policies based on evidence. The present study has some limitations. The first is related to the cross-sectional nature of the study, which prevented this study from establishing causation. Second, profiles of patients and environmental factors are likely to vary geographically, and, hence, the context in Shandong and Jiangsu may not be representative of all urban and rural areas in China. Third, the study was conducted in 2014, and may not entirely applicable to the present situation in China. Longitudinal studies with a large sample size are expected to further explore these questions.

## Conclusions

In this study, we found that the community health service for chronic disease management was not fully utilized in China. Determinants of infrequent community visits in diabetes patients include urban residence, lower household income, lack of health insurance, lack of telephone follow-up, and lack of household visit services. From the patients’ perspective, the reasons for infrequent visits in urban patients are drug scarcity and longer travel time to clinics. In rural patients, drug scarcity and concerns on medical expenses and are the most common barriers to clinic visits. Policy should be implemented to make community health care more accessible and affordable. The ministry of health and primary health care providers should develop both tools for behaviour change and weight management tools and services that are affordable and convenient in order to effectively improve diabetes patients’ self-management. In addition, a programme-specific training for health care workers is required to improve the quality of care. Finally, adequate supply of essential medicine in community health service and basic medic are, especially in rural areas, would contribute to more active primary care visits.

## Supplementary information

**Additional file 1: Table S1a.** Variable parameters associated with infrequent visitor status based on multiple logistic regression analyses. **Table S1b.** Variable parameters associated with infrequent visitor status based on multiple logistic regression analyses among urban adults. **Table S1c.** Variable parameters associated with infrequent visitor status based on multiple logistic regression analyses among rural adults.

## Data Availability

The raw dataset analyzed in the current study are available from the corresponding author on reasonable request.

## Abbreviations

CHWs: Community health workers
GP: General physician
T2DM: Type 2diabetes mellitus
