# Supplementary material for: Analysis of factors influencing the frequency of primary care visits among diabetic patients in two provinces in China
Source: BMC Public Health. 2019 Sep 13;19:1267. doi: 10.1186/s12889-019-7591-6 (PMC6743148; doi:10.1186/s12889-019-7591-6)
Supplement: Supplementary file 1 — Additional file 1: Table S1a. Variable parameters associated with infrequent visitor status based on multiple logistic regression analyses. Table S1b. Variable parameters associated with infrequent visitor status based on multiple logistic regression analyses among urban adults. Table S1c. Variable parameters associated with infrequent visitor status based on multiple logistic regression analyses among rural adults. [file 12889_2019_7591_MOESM1_ESM.docx]

# Table S1a. Variable parameters associated with infrequent visitor status based on multiple logistic regression analyses

| Variables | B | S.E, | Wals χ2 | OR  (95% CI) | P value |
| --- | --- | --- | --- | --- | --- |
| Urban vs. rural | .590 | .145 | 16.579 | 1.696 (1.293, 2.224) | < 0.001 |
| Male vs. female | -.004 | .119 | .001 | 0.996 (0.798, 1.257) | 0.972 |
| Age |  |  |  |  |  |
| ≤55yr |  |  | 5.532 | ─ |  |
| 56-65yr | -.380 | .163 | 5.459 | 0.684 (0.497,0.941) | 0.063 |
| ≥66yr | -.224 | .160 | 1.967 | 0.799 (0.585,1.093) | 0.161 |
| Medical history |  |  |  |  |  |
| Hypertension (yes vs. no) | -.291 | .115 | 6.461 | 0.747 (0.597,0.936) | 0.061 |
| Myocardial infarction (yes vs. no) | -.330 | .471 | .488 | 0.719 (0.285,1.812) | 0.485 |
| Stenting (yes vs. no) | -.038 | .760 | .003 | 0.963 (0.217,4.267) | 0.960 |
| CABG (yes vs. no) | .799 | .720 | 1.232 | 2.223 (0.542,9.116) | 0.267 |
| Stroke (yes vs. no) | -.070 | .221 | .100 | 0.932 (0.605,1.438) | 0.751 |
| Educational level, n (%) |  |  |  |  |  |
| Intermediate school or lower |  |  | .944 | ─ |  |
| High school | .019 | .183 | .011 | 1.019(0.711, 1.459) | 0.918 |
| ≥College graduate | .277 | .286 | .938 | 1.319(0.753,2.313) | 0.333 |
| Per capita household income |  |  |  |  |  |
| ≥20000 |  |  | 23.367 | ─ |  |
| 5000~19999 | .500 | .152 | 10.825 | 2.008 (1.487, 2.712) | < 0.001 |
| < 5,000 | .841 | .174 | 23.258 | 2.621 (1.859,3.696) | < 0.001 |
| Lack health insurance, (yes vs. no) | 1.839 | .523 | 12.361 | 6.854 (1.992, 23.578) | 0.002 |
| Household visit (yes vs. no) | -1.209 | .137 | 78.129 | 0.313 (0.241, 0.407) | < 0.001 |
| Telephone follow-up (yes vs. no) | -.663 | .123 | 29.057 | 0.507 (0.400, 0.643) | < 0.001 |

# Table S1b. Variable parameters associated with infrequent visitor status based on multiple logistic regression analyses among urban adults

| Variables | B | S.E, | Wals χ2 | OR  (95% CI) | P value |
| --- | --- | --- | --- | --- | --- |
| Male vs. female | .074 | .165 | .202 | 1.077(0.780,1.487) | 0.653 |
| Age |  |  |  |  |  |
| ≤55yr |  |  | 1.606 | ─ |  |
| 56-65yr | -.303 | .239 | 1.605 | 0.739 (0.463,1.180) | 0.205 |
| ≥66yr | -.205 | .229 | .802 | 0.814 (0.519,1.277) | 0.370 |
| Medical history |  |  |  |  |  |
| Hypertension (yes vs. no) | -.132 | .161 | .670 | 0.877 (0.639,1.202) | 0.413 |
| Myocardial infarction (yes vs. no) | -.103 | .571 | .032 | 0.902 (0.294,2.766) | 0.857 |
| Stenting (yes vs. no) | -1.943 | 1.186 | 2.683 | 0.143 (0.014,1.465) | 0.101 |
| CABG (yes vs. no) | 2.720 | 1.486 | 3.348 | 15.175 (0.824,279.428) | 0.067 |
| Stroke (yes vs. no) | -.212 | .289 | .539 | 0.809 (0.459,1.425) | 0.463 |
| Educational level, n (%) |  |  |  |  |  |
| Intermediate school or lower |  |  | 1.062 | ─ |  |
| High school | .039 | .213 | .033 | 1.039 (0.685,1.577) | 0.856 |
| ≥College graduate | .302 | .293 | 1.059 | 1.352 (0.761,2.403) | 0.304 |
| Per capita household income |  |  |  |  |  |
| ≥20000 |  |  | 15.862 | ─ |  |
| 5000~19999 | .600 | .180 | 11.139 | 2.329 (1.638, 3.313) | < 0.001 |
| < 5,000 | .772 | .245 | 9.894 | 2.639 (1.616, 4.310) | < 0.001 |
| Lack health insurance, (yes vs. no) | 1.453 | .656 | 4.913 | 4.750(1.334, 16.912) | 0.027 |
| Household visit (yes vs. no) | -1.109 | .221 | 25.128 | 0.341 (0.222, 0.523) | < 0.001 |
| Telephone follow-up (yes vs. no) | -.561 | .171 | 10.693 | 0.604 (0.438, 0.834) | 0.004 |

# Table S1c. Variable parameters associated with infrequent visitor status based on multiple logistic regression analyses among rural adults

| Variables | B | S.E, | Wals χ2 | OR  (95% CI) | P value |
| --- | --- | --- | --- | --- | --- |
| Male vs. female | -.118 | .176 | .451 | 0.888 (0.629,1.255) | 0.502 |
| Age |  |  |  |  |  |
| ≤55yr |  |  | 3.197 | ─ |  |
| 56-65yr | -.406 | .229 | 3.122 | 0.667 (0.425,1.045) | 0.077 |
| ≥66yr | -.216 | .231 | .877 | 0.805 (0.512,1.267) | 0.349 |
| Medical history |  |  |  |  |  |
| Hypertension (yes vs. no) | -.459 | .167 | 7.529 | 0.632 (0.455,0.877) | 0.057 |
| Myocardial infarction (yes vs. no) | -20.127 | 13896.865 | .000 | 0.000 | 0.999 |
| Stenting (yes vs. no) | 41.605 | 25872.631 | .000 | 1.172E18 | 0.999 |
| CABG (yes vs. no) | -20.449 | 21957.432 | .000 | 0.000 | 0.999 |
| Stroke (yes vs. no) | .125 | .342 | .134 | 1.133 (0.580,2.214) | 0.714 |
| Educational level, n (%) |  |  |  |  |  |
| Intermediate school or lower |  |  |  | ─ |  |
| High school | -.004 | .383 | .000 | 0.996 (0.470,2.109) | 0.992 |
| Per capita household income |  |  |  |  |  |
| ≥20000 |  |  | 7.556 | ─ |  |
| 5000~19999 | .235 | .328 | .514 | 1.215 (1.027, 2.318) | < 0.001 |
| < 5,000 | .676 | .340 | 3.961 | 1.725 (1.196, 3.299) | < 0.001 |
| Lack health insurance, (yes vs. no) | 2.282 | .844 | 7.305 | 9.317(1.768, 49.091) | 0.008 |
| Household visit (yes vs. no) | -1.254 | .177 | 50.467 | 0.278 (0.198, 0.391) | < 0.001 |
| Telephone follow-up (yes vs. no) | -.794 | .183 | 18.856 | 0.432 (0.303, 0.615) | < 0.001 |
